# Targeting Androgen Receptor/Src Complex Impairs the Aggressive Phenotype of Human Fibrosarcoma Cells

**DOI:** 10.1371/journal.pone.0076899

**Published:** 2013-10-09

**Authors:** Gabriella Castoria, Pia Giovannelli, Marzia Di Donato, Ryo Hayashi, Claudio Arra, Ettore Appella, Ferdinando Auricchio, Antimo Migliaccio

**Affiliations:** 1 Department of Biochemistry, Biophysics, and General Pathology, 2^nd^ University of Naples, Naples, Italy; 2 Istituto per la Diagnosi e la Cura dei Tumori Fondazione “G. Pascale”, Naples, Italy; 3 Laboratory of Cell Biology, National Cancer Institute, Bethesda, Maryland, United States of America; Dresden University of Technology, Germany

## Abstract

**Background:**

Hormones and growth factors influence the proliferation and invasiveness of human mesenchymal tumors. The highly aggressive human fibrosarcoma HT1080 cell line harbors classical androgen receptor (AR) that responds to androgens triggering cell migration in the absence of significant mitogenesis. As occurs in many human cancer cells, HT1080 cells also express epidermal growth factor receptor (EGFR).

**Experimental:**

**Findings**: We report that the pure anti-androgen Casodex inhibits the growth of HT1080 cell xenografts in immune-depressed mice, revealing a novel role of AR in fibrosarcoma progression. In HT1080 cultured cells EGF, but not androgens, robustly increases DNA synthesis. Casodex abolishes the EGF mitogenic effect, implying a crosstalk between EGFR and AR. The mechanism underlying this crosstalk has been analyzed using an AR-derived small peptide, S1, which prevents AR/Src tyrosine kinase association and androgen-dependent Src activation. Present findings show that in HT1080 cells EGF induces AR/Src Association, and the S1 peptide abolishes both the assembly of this complex and Src activation. The S1 peptide inhibits EGF-stimulated DNA synthesis, cell matrix metalloproteinase-9 (MMP-9) secretion and invasiveness of HT1080 cells. Both Casodex and S1 peptide also prevent DNA synthesis and migration triggered by EGF in various human cancer-derived cells (prostate, breast, colon and pancreas) that express AR.

**Conclusion:**

This study shows that targeting the AR domain involved in AR/Src association impairs EGF signaling in human fibrosarcoma HT1080 cells. The EGF-elicited processes inhibited by the peptide (DNA synthesis, MMP-9 secretion and invasiveness) cooperate in increasing the aggressive phenotype of HT1080 cells. Therefore, AR represents a new potential therapeutic target in human fibrosarcoma, as supported by Casodex inhibition of HT1080 cell xenografts. The extension of these findings in various human cancer-derived cell lines highlights the conservation of this process across divergent cancer cells and identifies new potential targets in the therapeutic approach to human cancers.

## Introduction

Reports on the number of non-reproductive and human cancer cells expressing steroid receptors (SRs) are continuously increasing. Human pancreas cancer cells express AR and undergo cell motility upon AR phosphorylation induced by interleukin 6 [1]. Human colon carcinoma Caco-2 cells express estradiol receptor alpha (ER alpha) and respond to estradiol with activation of the Src tyrosine kinase and proliferation. Anti-estrogens inhibit these effects [2]. Estrogens play a role in lung carcinogenesis as initially suggested by the greater adverse effect of tobacco smoke in women as well as expression of ER (beta and alpha) in both human non-small cell lung cancer cells and primary cultures of normal bronchial epithelium. Estrogens stimulate growth of the non-small cell lung tumor line xenografts in mice and the pure anti-estrogen ICI 182,780 blocks this effect [3]. Additional findings on the role of estrogens in human lung cancer development have also been reported [4]. Taken together, these studies are attracting an increasing interest on the role of SRs in human proliferative diseases because of their potential therapeutic implications.

Epidemiological studies suggest that age and sex influence the natural history of human mesenchymal tumors ([5] and *refs therein*). Previously, clinical studies reported that SRs are expressed in a large set of human soft tissue sarcomas (STS) of different histological origin. AR is more frequently expressed in male STS, and females with sarcomas have a better prognosis than males. Additionally, menopause, pregnancy and postpartum state alter the growth of aggressive fibromatoses, thus indicating that hormonal milieu and growth factors influence the proliferation and spreading of STS [5]. However, the low survival rate in STS patients reflects the inadequacy of current therapies for these tumors and the need for new treatment strategies [6]. 

We recently discovered that human fibrosarcoma-derived HT1080 cells harbor classical, transcriptional incompetent AR, which mediates androgen-elicited migration, but not DNA synthesis [7]. Thus, the HT1080 cell line represents a new model of hormone-responsive cancer cells useful to dissect the role of androgen signaling independently of AR transcriptional activity. In different cell types, androgens act through a non-transcriptional mechanism by activating extra-nuclear circuits [8,9]. Thus, signaling effectors (ie, Src tyrosine kinase) [10,11] or scaffolds (ie, filamin A) [7] transfer mitogenic hormonal message to nuclei and/or increase cell motility by modifying cytoskeleton actin when they are directly engaged by a sub-population of extra-nuclear AR.

EGFR is frequently overexpressed and/or activated in STS-derived cells and tumors [12] and EGFR blockade has been proposed as a therapeutic strategy in mesenchymal-derived tumors [13]. We previously showed that EGF-coupled EGFR engages extra-nuclear AR to transmit its mitogenic signaling [14]. Indeed, AR mediates EGF proliferative activity in various cancer cells [15,16]. Therefore, it is conceivable that EGF/AR crosstalk plays a role in cancer development, when androgen levels decline as a consequence of ageing or pathological conditions. 

In this study, we report that the pure androgen antagonist Casodex inhibits the growth of HT1080 cells *in vivo*. A putative mechanism responsible for this effect is analyzed. Findings in this paper reveal that EGF transduces its signal through the AR/Src complex in HT1080 and various cancer-derived cell types, including colon and pancreatic cancer cells. In these cells, such a crosstalk regulates different biological responses that are crucial for tumor progression.

## Results

**Figure 1:**
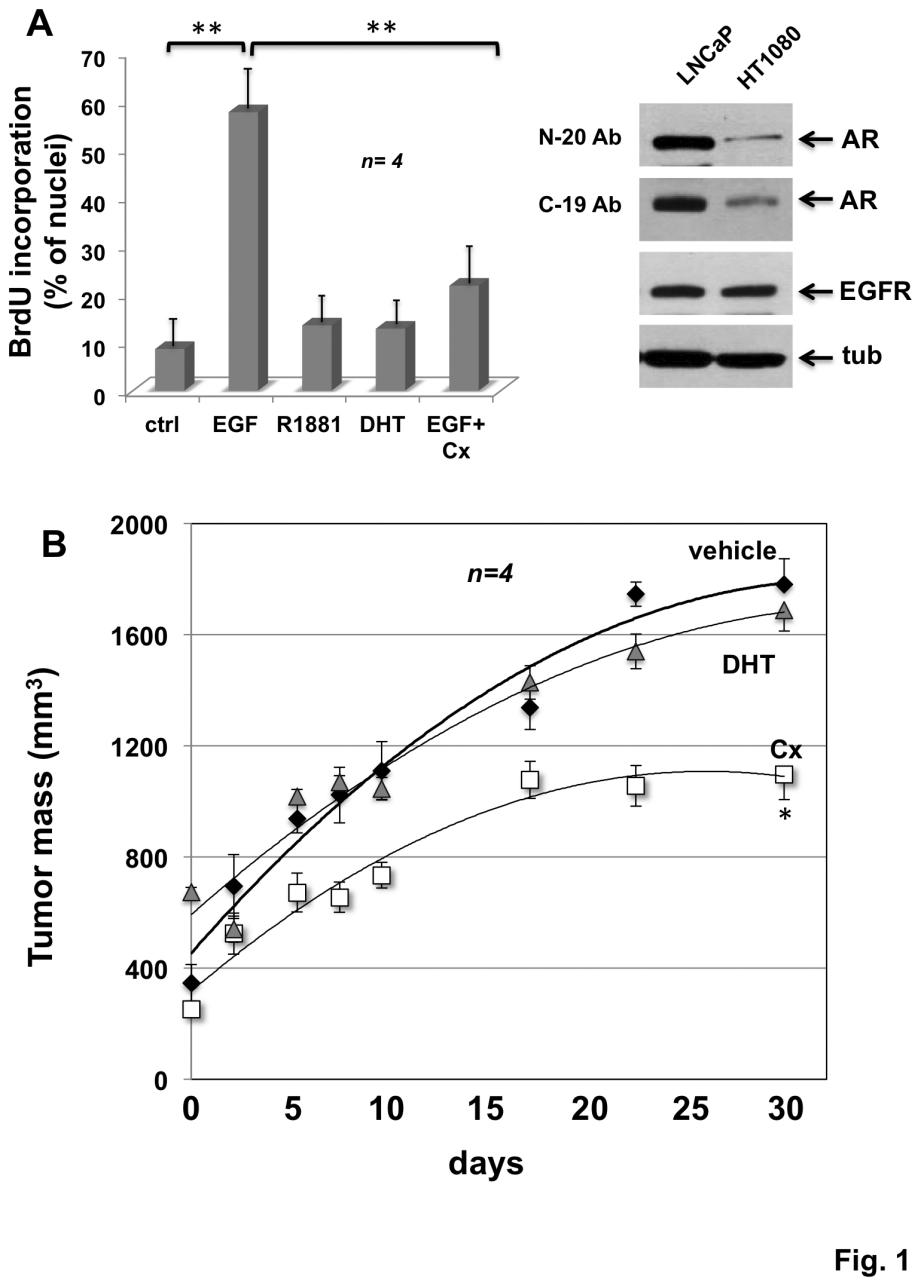
Casodex inhibits the EGF-triggered DNA synthesis in human fibrosarcoma HT1080 cells and reduces the growth of HT1080 xenografts. Human fibrosarcoma HT1080 cells were used. In **A**, quiescent cells on coverslips were left un-stimulated or stimulated for 18 h with the indicated compounds. EGF (Roche) was used at 100 ng/ml; the synthetic androgen R1881 and DHT (both from Sigma) were used at 10 nM; Casodex (Sigma) was used at 10 μM. After *in* *vivo* pulse with BrdU (100 μM; Sigma), BrdU incorporation was analyzed by IF and expressed as % of total nuclei. Several independent experiments were performed in duplicate and the results were derived from at least 400 scored cells for each coverslip. Mean and SEM are shown. n represents the number of experiments. (**) p value< 0,001. Inset in **A**, shows the Western blot of HT1080 cell lysates with the antibodies against the indicated proteins. AR was revealed using two different antibodies raised against the AR amino (N-20) or the carboxyl (C-19)-terminal domains of the receptor. Tubulin (tub) was revealed by immunoblot, as a loading control. In **B**, xenografts were established in nude male mice as described in Methods. On alternate days, animals were intra-peritoneally injected with vehicle alone (vehicle), or the pure androgen antagonist Casodex (Cx: 0.1 μg/mice; 4µg/Kg body weight), or DHT (DHT: 0,1 μg/mice; 3 μg/Kg body weight) alone. Volumes of HT1080 cell xenografts were measured at the indicated times in two dimensions by a caliper and expressed as tumor mass (mm^3^). Mean and SEM are shown. n represents the number of experiments. (*) p value < 0,05.

Human fibrosarcoma-derived HT1080 cells harbor a classical AR (inset in Figure 1A, [7]) that neither activates transcription in ARE-luc gene reporter assay, nor trans-locates into nuclei upon androgen stimulation of quiescent cells (Figure S1, A and B; [7]). As it has been observed in mouse embryo NIH3T3 fibroblasts [7,11], AR expressed in HT1080 cells mediates motility (Figure S1, [7]), but is unable to mediate DNA synthesis upon cell challenging with the synthetic androgen R1881 or DHT (Figure 1A). In contrast, HT1080 cells that express high level of EGFR (inset in Figure 1A) significantly incorporate BrdU into newly synthesized DNA upon stimulation with EGF. Remarkably, the pure androgen antagonist, Casodex inhibits the EGF-induced BrdU incorporation (Figure 1A). 

On the basis of the failure of R1881 or DHT to stimulate DNA synthesis and Casodex inhibition of EGF mitogenic effect, we investigated the effect of Casodex on growth of HT1080 cells using a mouse model of tumorigenesis. To this end, HT1080 xenografts were established in male immune-depressed mice and their growth was monitored for 4 weeks (Figure 1B). During this time frame, tumor mass significantly increased in vehicle-treated mice (vehicle), and treatment with DHT slightly affected this increase (Figure 1B). In contrast, tumor growth was drastically reduced in mice treated with Casodex (Cx; see the legend to the Figure 1). Therefore, the findings reported in Figure 1 collectively indicate that targeting AR with a largely used AR antagonist inhibits DNA synthesis induced by EGF in cultured HT1080 cells and impairs growth of tumor xenografts. To date, this is the first evidence that targeting AR inhibits human fibrosarcoma cell growth.

**Figure 2:**
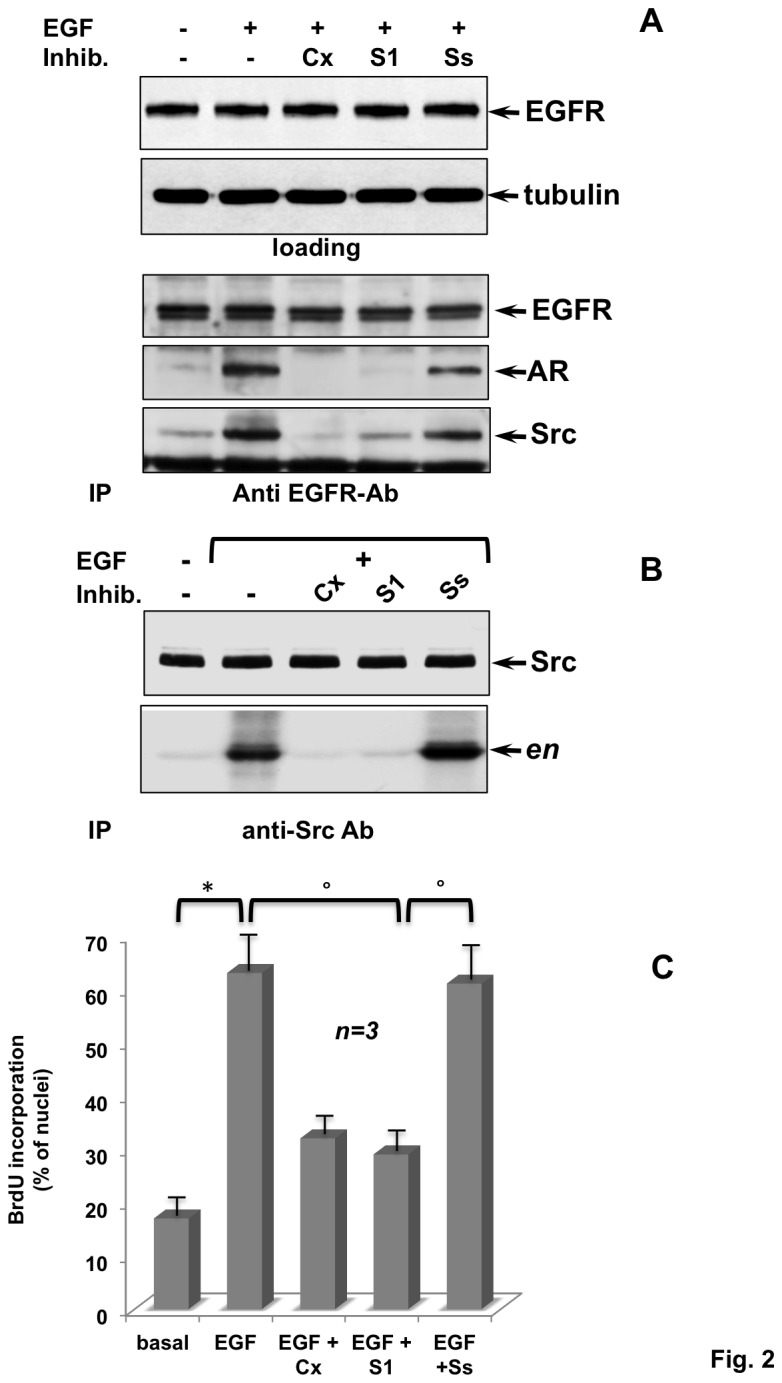
Inhibition by the S1 peptide of AR/Src complex, Src activation and DNA synthesis triggered by EGF in HT1080 cells. Quiescent HT1080 cells were used. Cells were left un-stimulated or stimulated for 10 min with EGF (at 100 ng/ml) in the absence or presence of S1 or Ss peptide (both at 1 nM). Casodex (at 10 μM) was used for comparison with the S1 peptide. Upper section in **A**, Western blot of HT1080 cell lysates with anti-EGFR antibody. Tubulin (tubulin) was revealed by immunoblot, as a loading control. Lower section in **A**, lysates were immune-precipitated with anti-EGFR Ab and proteins in immune complexes were detected using antibodies against the indicated proteins. In **B**, lysates were immune-precipitated with the anti-Src MAb and Src activity in immune complexes was assayed using acidified enolase, as a substrate. In **C**, cells on coverslips were left untreated or treated for 18 h with the indicated compounds. After *in* *vivo* pulse with BrdU (100 μM), BrdU incorporation was analyzed by IF and expressed as % of total nuclei. Several independent experiments were performed in duplicate and the results were obtained from at least 500 scored cells for each coverslip. Mean and SEM are shown. n represents the number of experiments. (*) p value < 0,001; (°) p value< 0,005.

We then verified the hypothesis that inhibition of DNA synthesis and HT1080 cell growth by Casodex is caused by its interference in the EGF/AR crosstalk. Such a crosstalk has been previously analyzed in prostate cancer-derived LNCaP cells [15]. To this end, we used a small proline-rich peptide, the S1 peptide, which rapidly diffuses across membranes and functions at nano-molar concentration in various cell types [16], including HT1080 cells (Figure S1). The S1 peptide derives from the 377-386 amino-acid sequence of AR that is rich in prolines and is responsible for association of AR with the Src-SH3 domain. The S1 peptide competes for this association in androgen-challenged prostate cancer LNCaP cells [16]. Based on these findings, we verified whether the peptide inhibits AR/Src association in HT1080 cells. First, in co-immune-precipitation experiment we observed that EGF stimulation of quiescent HT1080 cells induces association of EGF-R with AR and Src (lower panel in Figure 2A). In addition to disrupting AR/Src Association, the S1 peptide abolishes AR/Src association with EGF-R. Such recruitment likely depends on Src activation of the AR/Src complex [15]. In agreement with this hypothesis, Figure 2B shows that 1 nM S1 peptide completely abolishes Src activity stimulation triggered by EGF in quiescent HT1080 cells. This inhibition is comparable to that observed by treatment of cells with 10 μM Casodex. Lastly, the S1 peptide abolishes the mitogenic response elicited by EGF, as shown by BrdU incorporation analysis in Figure 2C. Again, this inhibition is comparable to that exerted by Casodex. These findings indicate that mitogenic signaling of EGF requires EGFR/AR/Src complex assembly in HT1080 cells. Of note, the effect of the S1 peptide is specific, since the scrambled peptide (Ss peptide), having the same composition as the S1 peptide, but a completely different sequence [16], only slightly affects the EGFR/AR/Src complex assembly (Figure 2A) as well as Src activation (Figure 2B) and DNA synthesis (Figure 2C) triggered by EGF in HT1080 cells.

Findings in Figure 2 show that EGF stimulates DNA synthesis through EGFR/AR/Src association and Src activation in HT1080 cells. By competing for AR/Src Association, the S1 peptide prevents the EGF mitogenic signaling in fibrosarcoma cells.

**Figure 3:**
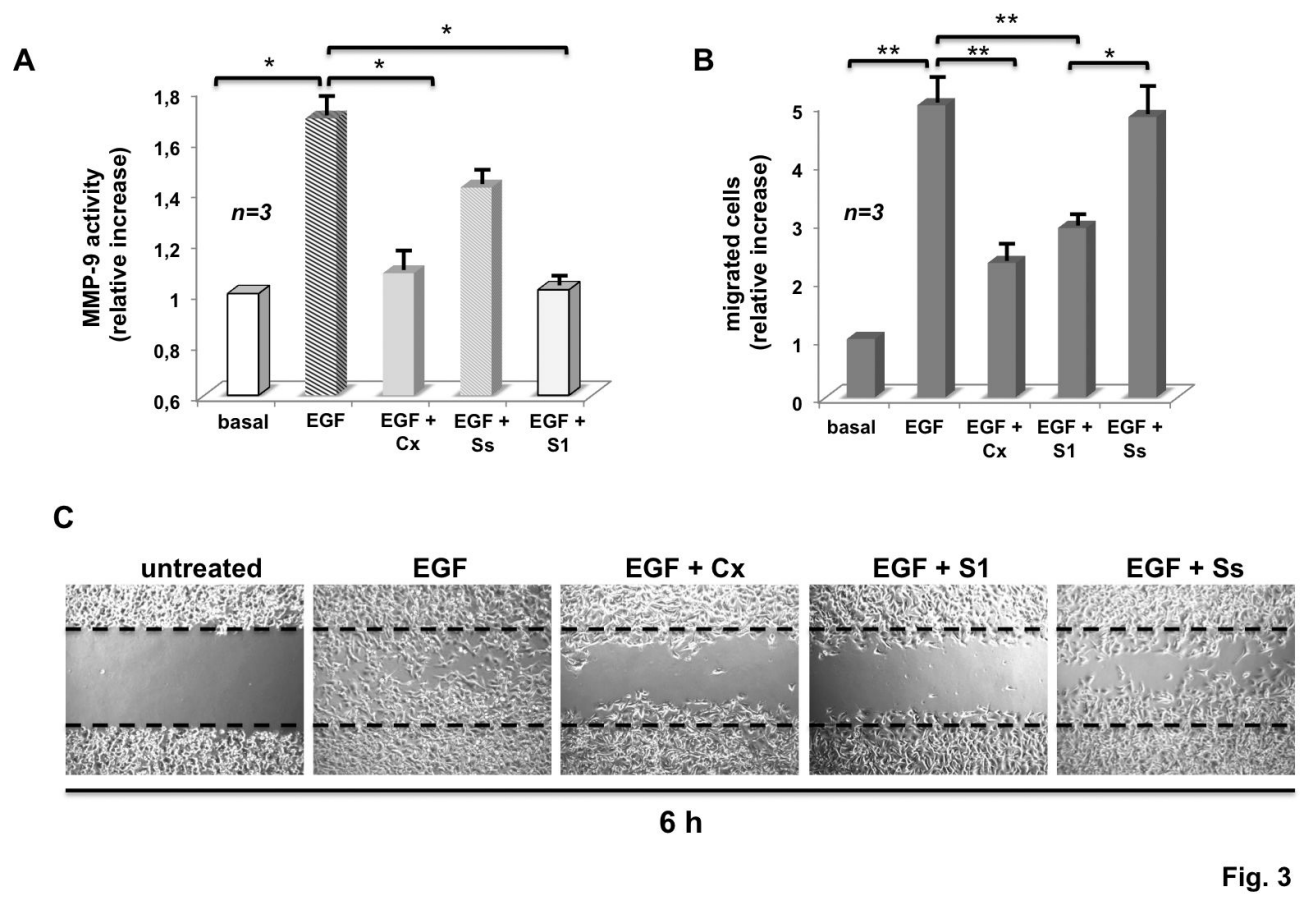
S1 peptide inhibits EGF-stimulated MMP-9 secretion, transmigration and wound closure in HT1080 cells. Quiescent HT1080 cells were used. In **A**, the cells were left untreated or treated for 5 min with EGF (at 100 ng/ml), in the absence or presence of Casodex (Cx, at 10 μM), S1 or SS peptides (both peptides were used at 10 nM). MMP-9 protease activity was assayed in concentrated conditioned cell medium, as detailed in Methods. In B and C, the cells were left untreated or treated for 6 h with the indicated compounds. EGF was used at 100 ng/ml; Casodex (Cx) was used at 10 μM; both S1 and SS peptides were used at 10 nM. In **B**, cells were allowed to migrate in collagen pre-coated Trans-well filters. Migrated cells were stained and counted as reported in Methods In A and B, results were derived from several independent experiments, each performed in duplicate. Data are expressed as relative increase. Mean and SEM are shown. n represents the number of experiments. In A and B, (*) *p* value < 0.005; (**) *p* value < 0.001. In **C**, contrast phase images from wounded cells were captured and shown. They are representative of 3 different experiments, each performed in duplicate.

Cancer progression and invasiveness include migration-associated proteolytic interaction of cancer cells with extra-cellular matrix (ECM). This process requires expression and activation of matrix metallo-proteinases (MMPs) [17]. MMP-9 activation and release have been previously studied in HT1080 cells [18]. In Figure 3A, we observe by that EGF increases by about 2-fold the protease activity of MMP-9 released from quiescent HT1080 cells and Casodex inhibits this response (Figure 3A). Here again, the S1 peptide inhibits the EGF effect on MMP-9 secretion (Figure 3A). The Ss peptide, used as a control, showed a negligible inhibitory effect (Figure 3A and legend to this Figure). EGF increases by 5 fold the invasiveness of HT1080 cells in trans-migration assay and this effect is inhibited to a similar extent by Casodex and S1 peptide, but not by Ss peptide (Figure 3B). Lastly, quiescent HT1080 cells were wounded and allowed to migrate in the absence or presence of the compounds indicated in Figure 3C. Contrast-phase images show that EGF induces an almost complete wound closure. Only few cells migrate upon treatment with Casodex, supporting the view that AR is also implicated in the migratory phenotype triggered by EGF. The S1 peptide consistently inhibits the EGF-induced effect, whereas the Ss peptide shows a much weaker inhibitory effect. Control images captured from untreated cells are shown for comparison. 

Altogether, these findings indicate that EGFR/AR/Src complex regulates motility and invasiveness induced by EGF in HT1080 cells. In addition to inhibiting the proliferative response, Casodex and the S1 peptide also impair the migratory phenotype induced by EGF in these cells.

**Figure 4:**
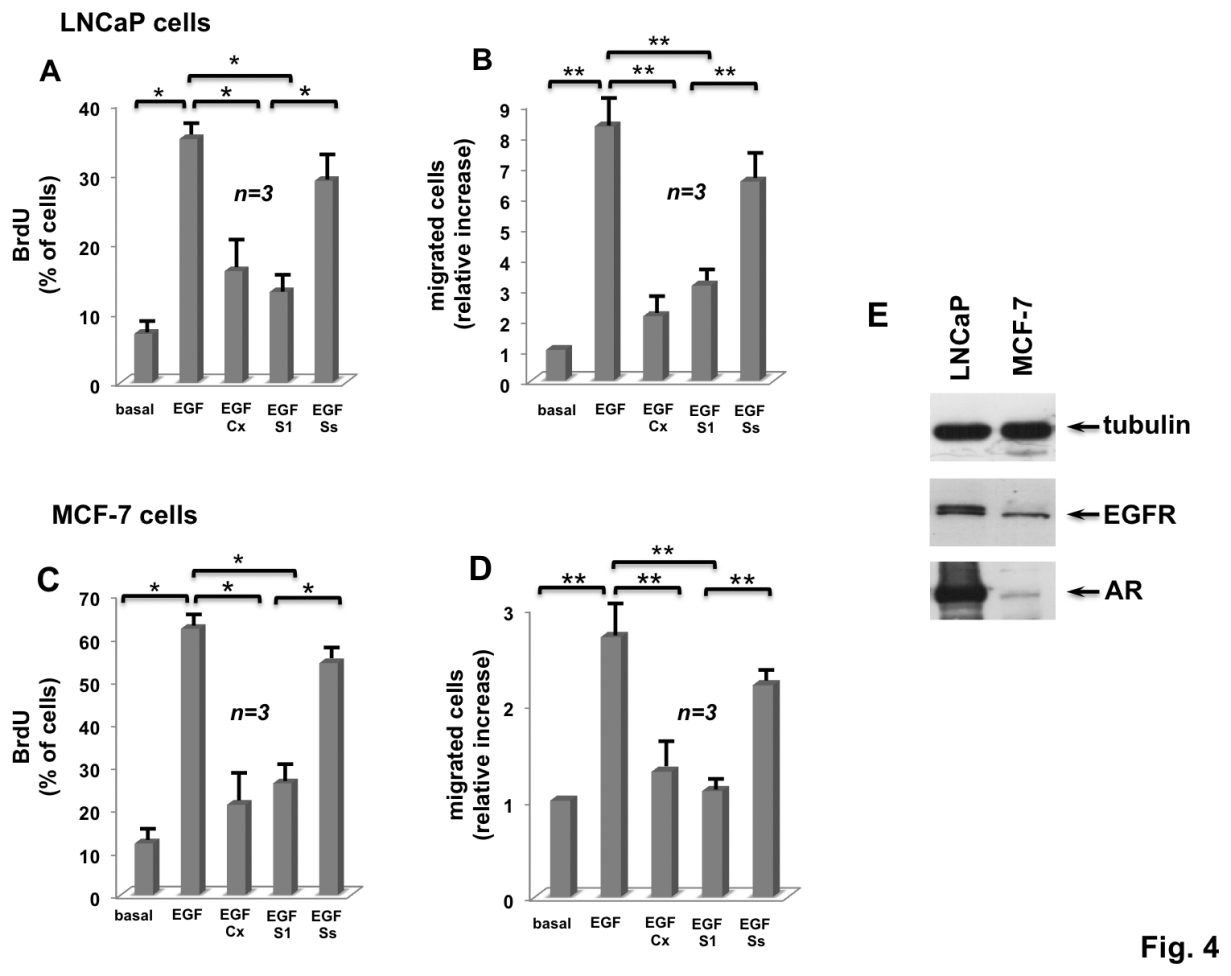
Casodex and S1 peptide inhibit EGF-stimulated BrdU incorporation and migration in LNCaP and MCF-7 cells. Quiescent human prostate cancer-derived LNCaP cells were used. In **A**, quiescent cells on coverslips were left unstimulated or stimulated for 18 h with the indicated compounds. EGF was used at 100 ng/ml; Casodex was used at 10 μM; S1 and Ss peptides were used at 10 nM. After *in* *vivo* pulse with BrdU (100 μM), BrdU incorporation was analyzed by IF and expressed as % of total cells. Several independent experiments were performed in duplicate and the results were derived from at least 600 scored cells for each coverslip. Mean and SEM are shown. n represents the number of experiments. In **B**, the cells were left untreated or treated for 6 h with the indicated compounds. EGF was used at 100 ng/ml; Casodex (Cx) was used at 10 μM; both S1 and SS peptides were used at 1 nM. Cells were allowed to migrate in collagen pre-coated Trans-well filters. Migrated cells were stained and counted as reported in Methods. Results were derived from several independent experiments, each performed in duplicate. Data are expressed as relative increase. Mean and SEM are shown. n represents the number of experiments. Quiescent human breast cancer-derived MCF-7 cells were used. In **C**, quiescent cells on coverslips were left un-stimulated or stimulated for 18 h with the indicated compounds. EGF was used at 100 ng/ml; Casodex was used at 10 μM; S1 and Ss peptides were used at 1nM. After *in* *vivo* pulse with BrdU (100 μM), BrdU incorporation was analyzed by IF and expressed as % of total cells. Several independent experiments were performed in duplicate and the results were derived from at least 500 scored cells for each coverslip. Mean and SEM are shown. n represents the number of experiments. In **D**, the cells were left untreated or treated for 6 h with the indicated compounds. EGF was used at 100 ng/ml; Casodex (Cx, at 10 μM); both S1 and SS peptides were used at 10 nM. Cells were allowed to migrate in collagen-pre-coated Trans-well filters. Migrated cells were stained and counted as reported in Methods. Results were derived from several independent experiments, each performed in duplicate. Data are expressed as relative increase. Mean and SEM are shown. n represents the number of experiments. In **A**, B, C and **D**, (*) *p* value < 0.001; *p* value < 0,005 (**). Panel **E** shows the Western blot of LNCaP or MCF-7 cell lysates with the antibodies against the indicated proteins: tubulin, epidermal growth factor receptor (EGFR) and androgen receptor (AR).

Various untransformed and cancer-derived cell types express both AR and EGFR [15,16]. We therefore extended our analysis to quite divergent cancer cells. We first used classical hormone-responsive cells derived from human prostate (LNCaP cells) and mammary (MCF-7 cells) cancers. These two steroid-responsive cell lines are considered classical, *reproductive* cells and express both AR and EGFR (panel E in Figure 4) [15]. The two cell lines (Figure 4A and C) robustly incorporate BrdU into newly synthesized DNA upon stimulation with EGF. Casodex inhibits EGF-induced BrdU incorporation and S1 peptide also abolishes the mitogenic response elicited by EGF. Such inhibition is almost comparable to that exerted by Casodex. Here again, the effect of the S1 peptide is specific, since the Ss peptide, used as a control, only slightly affects DNA synthesis triggered by EGF in these cells. Consistent with findings in HT1080 cells, EGF significantly increases the motility of LNCaP (Figure 4B) and MCF-7 (Figure 4D) cells in transmigration assay. This effect is inhibited to a similar extent by Casodex and S1 peptide, but not by Ss peptide (Figure 4B and D). 

This set of experiments indicates that both mitogenic and migratory signaling of EGF requires AR/Src complex assembly in human prostate and mammary cancer-derived cells.

Up to now, scant evidence has been accumulated as to the role of AR and its crosstalk with EGF signaling in so-called non-classical, *non-reproductive* cells. To this end, we first used untransformed NIH3T3 fibroblasts. These cells harbor a transcriptionally inactive AR (Figure S2, panel A) [11] that mediates motility (Figure S1 and ref. 7), but not cell proliferation upon androgen stimulation (Figure S2, panel B) [11]. They also express EGFR (Figure S2, panel C, inset). Stimulation of NIH3T3 cells with EGF significantly increases BrdU incorporation (Figure S2, panel C) and motility (Figure S2, panel D) of these cells. Casodex and S1 peptide significantly inhibit both the responses triggered by EGF (Figure S2, C and D). The control Ss peptide slightly modifies DNA synthesis (Figure S2, panel C) and migration (Figure S2, panel D) of NIH3T3 cells challenged with EGF.

**Figure 5:**
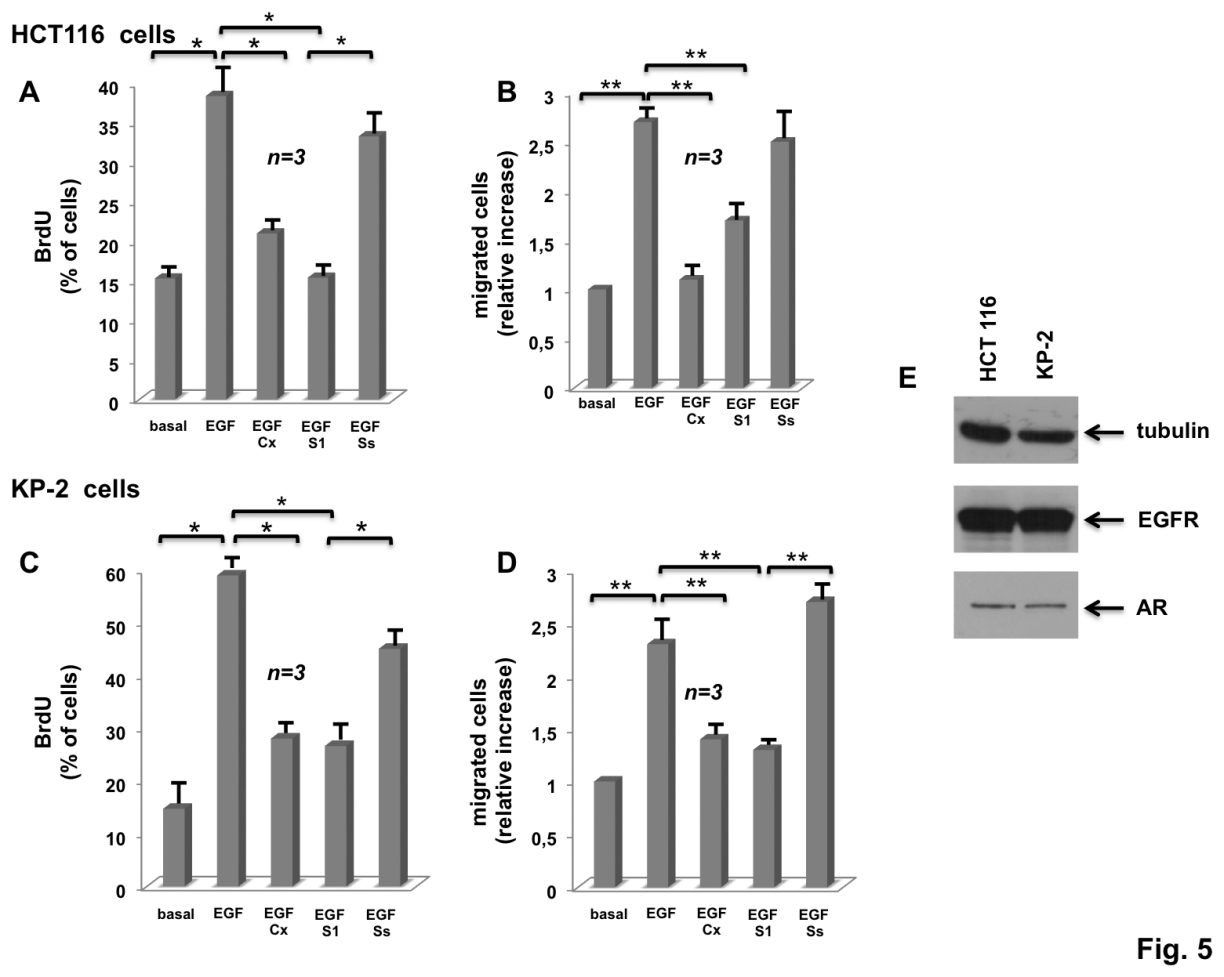
Casodex and S1 peptide inhibit EGF-stimulated BrdU incorporation and migration in HCT116 and KP-2 cells. Quiescent human colon cancer-derived HCT116 cells were used. In **A**, cells on coverslips were left un-stimulated or stimulated for 18 h with the indicated compounds. EGF was used at 100 ng/ml; Casodex was used at 10 μM; S1 and Ss peptides were used at 10 nM. After *in* *vivo* pulse with BrdU (100 μM), BrdU incorporation was analyzed by IF and expressed as % of total cells. Several independent experiments were performed in duplicate and the results were derived from at least 400 scored cells for each coverslip. Mean and SEM are shown. n represents the number of experiments. In **B**, the cells were left untreated or treated for 6 h with the indicated compounds. EGF was used at 100 ng/ml; Casodex (Cx) was used at 10 μM; both S1 and SS peptides were used at 10 nM. Cells were allowed to migrate in collagen-pre-coated Trans-well filters. Migrated cells were stained and counted as reported in Methods. Results were derived from several independent experiments, each performed in duplicate. Data are expressed as relative increase. Mean and SEM are shown. n represents the number of experiments. Quiescent human pancreatic cancer-derived KP-2 cells were used. In **C**, cells on coverslips were left unstimulated or stimulated for 18 h with the indicated compounds. EGF was used at 100 ng/ml; Casodex was used at 10 μM; S1 and Ss peptides were used at 10 nM. After *in* *vivo* pulse with BrdU (100 μM), BrdU incorporation was analyzed by IF and expressed as % of total cells. Several independent experiments were performed in duplicate and the results were derived from at least 400 scored cells for each coverslip. Mean and SEM are shown. n represents the number of experiments. In **D**, the cells were left untreated or treated for 6 h with the indicated compounds. EGF was used at 100 ng/ml; Casodex (Cx) was used at 10 μM; both S1 and SS peptides were used at 10 nM. Cells were allowed to migrate in collagen-pre-coated Trans-well filters. Migrated cells were stained and counted as reported in Methods. Results were derived from several independent experiments, each performed in duplicate. Data are expressed as relative increase. Mean and SEM are shown. n represents the number of experiments. In **A**, B, C and **D**, (*) *p* value < 0.001; (**) p value < 0,005. Panel **E** shows the Western blot of HCT116 or KP-2 cell lysates with the antibodies against the indicated proteins: tubulin, epidermal growth factor receptor (EGFR) and androgen receptor (AR).

We next evaluated the effect of Casodex and S1 peptide on DNA synthesis and motility induced by EGF in human colon cancer-derived HCT116 cells. The Western blot in Figure 5 (panel E) shows that these cells do in fact express both AR and EGFR. Stimulation with EGF robustly increases BrdU incorporation (panel A) and motility (panel B) of these cells. Casodex and S1 peptide similarly inhibit these EGF-elicited responses (A and B in Figure 5). Lastly, we used human pancreatic cancer-derived KP-2 cells. Here again, the immunoblot of lysate proteins shows that both AR and EGFR are expressed in these cells (panel E in Figure 5). EGF challenging of KP-2 quiescent cells significantly increases BrdU incorporation (panel C) as well as motility assayed by transmigration analysis (panel D). The anti-androgen Casodex and S1 peptide inhibit both DNA synthesis and motility induced by EGF in these cells (panels A and B in Figure 5). The control Ss peptide does not interfere with the EGF-elicited responses in either HCT116 or KP-2 cells (panels A, B, C and D in Figure 5). 

In sum, this set of experiments for the first time shows that EGF signaling relies on AR/Src complex in colon and pancreatic cancer cells. AR or AR/Src blockade by Casodex or S1 peptide impairs the biological responses challenged by EGF in these cancer cells. 

## Discussion

Soft tissue sarcomas (STS) arise from mesenchymal tissues and surgery, radiotherapy, as well as chemotherapy have improved the survival rate in STS patients. Despite these treatments, however, STS are fatal for most patients and the survival rate still remains unsatisfactory. New therapeutic approaches are therefore needed for inhibition of mesenchymal cancer growth and metastasis ([6] and refs therein). 

In this article we investigated the role of AR in human fibrosarcoma HT1080 cells, based on our observation that these cells harbor a noticeable amount of transcriptional incompetent AR [7]. This receptor mediates motility, but not DNA synthesis upon androgen stimulation. In contrast, the HT1080 cells are strongly responsive for growth to many growth factors such as EGF. Unexpectedly, the anti-androgen Casodex inhibits not only the hormone effect but also the EGF-stimulated DNA synthesis. More interestingly, Casodex significantly impairs the growth of HT1080 cell xenografts. These findings indicate that classical AR is involved in the EGF-dependent HT1080 cell growth. A crosstalk between AR and EGFR was previously observed in human hormone-dependent prostate cancer-derived LNCaP cells [15]. In these cells, we dissected the mechanism of this crosstalk and discovered that Src tyrosine kinase provides the molecular link between EGF and androgen signaling. In fact, EGF induces association of EGFR, AR and Src and inhibition of Src/AR complex assembly by Casodex suppresses Src activation and mitogenesis induced by EGF [15]. In the present study, we observe similar findings and the consequent inhibitory effect of Casodex on HT1080 cell proliferation. 

We also analyzed the EGF/AR cross talk in HT1080 cells, using the S1 peptide that specifically targets AR/Src interaction. This peptide derives from the AR sequence responsible for association of a proline rich sequence of AR with Src-SH3 domain and competes for this association in androgen-challenged prostate cancer LNCaP cells [16]. Together with the ER alpha-derived peptide interfering in ER alpha/Src-SH2 domain association [19], the S1 peptide represents a prototype of a new category of receptor antagonists that target non-transcriptional action of SRs and leave unaffected their transcriptional action [16,19]. Such a selective inhibition should spare the positive effects dependent on receptor transcriptional action and therefore should be better tolerated by patients. 

The findings reported here on the effect of AR/Src complex inhibition on cell invasiveness clearly suggest new roles to be played by steroid receptors in cancers derived from non-hormone dependent tissues. The migratory potential of tumor cells is a crucial factor in tumor progression. This process, however, requires acquisition of an invasive phenotype involving MMPs production and cell motility [20]. Results in this paper show that transmigration and MMP-9 secretion from HT1080 cells are stimulated by EGF and inhibited at the same extent by both, Casodex and S1 peptide. Thus, AR/Src complex regulates multiple processes in EGF-stimulated HT1080 cells and led us to conclude that prevention of AR/Src Association, independent of the presence of androgens, impairs several properties that are crucial for cancer progression and invasiveness. 

In sum, AR antagonists or AR/Src complex inhibitors might be applied in the human STS clinical setting to reduce side effects of chemotherapy, a limiting factor in STS therapy. Our study shows that AR inhibition by Casodex results in significant antitumor activity against fibrosarcoma *in vitro* and *in vivo*. Thus, AR blockade, alone or in combination with other therapies, may represent a promising therapeutic intervention for the treatment of STS. Because of the heterogeneity of STS, clinical trials with AR antagonists would require appropriate patient selection, drug scheduling, and combination therapies. AR antagonists in combination with inhibitors of additional STS-molecular targets (e.g., EGFR and/or AR/Src complex) might emerge as a novel therapeutic strategy, since STS frequently exhibit derangements of EGFR and tyrosine kinase signaling ([12,13] and refs therein). 

Results obtained using *reproductive* and *non-reproductive* cells, untransformed cells (fibroblasts) or cells derived from different human cancers (prostate, breast, colon and pancreas) indicate that AR blockade by Casodex impairs EGF-elicited biological responses in these cells. These findings place our study in a more general context and deal with an aspect of AR biology and anti-androgen therapy that has not been effectively approached to date. It is conceivable that the increasing number of human cancers expressing SRs and responding to steroids and/or growth factors should in the near future increase the number of tumors that might benefit from advances in the therapy of classical hormone-dependent cancers. Lastly, findings obtained using the S1 peptide open up the possibility of developing new approaches in human cancers, since targeting protein/protein interactions is an emergent and very exciting field that will likely hold significant advances for cancer therapy. 

## Materials and Methods

### Cell culture

Human fibrosarcoma-derived HT1080 cells are a generous gift of Prof. P Friedl (Radboud University Nijmegen Medical Centre, 6500HC Nijmegen, The Netherlands). HT1080, mouse embryo NIH3T3 (ATCC- LGC Standards, Milan, Italy) and fast growing prostate cancer-derived LNCaP (ATCC) cells were cultured, made quiescent and transfected as reported [7,10,21]. Breast cancer-derived MCF-7 cells (ATCC) were cultured and made quiescent as reported [21]. Human colon carcinoma-derived HCT116 cells are a generous gift of Prof. L. Altucci (Department of Biochemistry, Biophysics and General Pathology- II University of Naples - Naples, Italy). They were cultured at 5% CO_2_ in phenol-red Dulbecco's modified Eagle's medium (DMEM) supplemented with 10% fetal bovine serum (FBS), pen/strepto (100 Units) and glutamine (2mM). The cells were made quiescent by serum starvation for 24 h. During this time frame, they were maintained in phenol red-free DMEM containing pen/strepto (100 Units) and glutamine (2 mM). Human pancreaatic cancer-derived KP-2 cells were from Health Science Research Resources Bank (HSRRB; Osaka 590-0535, Japan). They were cultured at 5% CO2 in phenol red RPMI-1640 (RPMI, Roswell Park Memorial Institute) medium supplemented with 10% FBS, pen/strepto (100 Units), glutamine (2 mM), sodium pyruvate (1mM) and non essential aminoacids (0.1 mM). The cells were made quiescent using phenol red-free RPMI-1640 supplemented with 0.1% FBS, pen/strepto (100 Units) and glutamine (2 mM) for 24 h. 

### Constructs and transactivation assay

cDNA encoding wild-type hAR was in pSG5 [22]. The 3416 construct, containing four copies of the wild-type *slp*-HRE2 (5’-TGGTCAgccAGTTCT-3’) and the 3424 construct (5’-TGGACAgccAGTTCT-3’), were cloned in the Nh*e*I site in pTK-TATA-Luc [23]. These constructs are a generous gift of Dr. F. Claessens (Molecular Endocrinology Laboratory, Department of Cellular and Molecular Medicine, KU Leuven, Campus Gasthuisberg, BE-3000 Leuven, Belgium). Transactivation assay in sub-confluent HT1080 and NIH3T3 cells was performed as reported [7,11], using phenol red–free DMEM containing 10% charcoal-stripped serum. Cells were transfected by Superfect with 2 μg of 3416-pTK-TATA-Luc or 3424-pTK-TATA-Luc plasmid, alone or with 1 μg pSG5- hAR–expressing plasmid. Twenty-four h later, transfected cells were left un-stimulated or stimulated with 10 nM R1881 (Perkin Elmer) for 18 h. Luciferase activity from lysates was measured using a luciferase assay system (Promega) and values corrected using CH110-expressed-β-galactosidase activity (GE Healthcare). Values were obtained from several independent experiments, each performed in triplicate. 

### Peptides and uptake analysis of labeled peptides

The Src-S1 (S1; Ac-PPPHPHARIK-NH2) and the scrambled (Ss; Ac-HPKPARIPHP-NH2) peptides were designed and synthesized as reported [16]. Both peptides were N-terminal acetylated and C-terminal amidated [16]. Unless otherwise stated, they were used at 10 nM. For uptake analysis of labeled peptides, exponentially growing HT1080 cells were dissociated with a non-enzymatic cell dissociation medium (Sigma). About 2.5 x 10^5^ cells were plated and cultured overnight on 30 mm plates on glass coverslips. The cells were then made quiescent. The medium was discarded, and the cells were washed with NaCl/Pi (pH 7.3). NaCl/Pi was discarded, and the cells were incubated with the S1 and the Ss peptides conjugated to 5-(6)-carboxyfluorescein succinimidyl ester (Molecular Probes). Fluorescein-conjugated peptides were dissolved in Opti-MEM and added (at 1 nM) at 4°C or 37°C for 30 min to the cell medium. For detection of fluorescein-labeled peptides, the cells were washed three times with NaCl/Pi before being fixed [21] and processed in Vectashield mounting medium (Vector Laboratories, Burlingame, CA, USA). The distribution of fluorescence in fixed cells was analyzed with a DMBL Leica (Leica) fluorescent microscope using HCX PL Apo 63x oil objective. Images were captured using DC480 camera (Leica) and acquired using FW4000 (Leica) software.

### BrdU incorporation

After *in vivo* labeling with 100 μM BrdU (Sigma), DNA synthesis was analyzed by immunofluorescence [21], using diluted (1:50 in PBS) mouse monoclonal anti-BrdU antibody (clone BU-1, from GE Healthcare). 

### Wound scratch, migration and matrix metalloproteinase-9 (MMP-9) assays

For wound scratch assay, quiescent HT1080 cell monolayers at confluence were wounded using sterile pipette tips, and then washed in PBS [7]. To avoid proliferation, cells were treated with cytosine arabinoside (at 100 μM; Sigma) and then left un-stimulated or stimulated for 6h with EGF (from Roche, at 100ng/ml). The anti-androgen, Casodex (Astra-Zeneca or Sigma) was used at 10 μM. Fields were analyzed with DMIRB inverted microscope (Leica) using N-Plan 10x objective (Leica). Contrast-phase images were captured using a DC200 camera (Leica) and acquired using IMI1000 (Leica) software, as previously described [7]. Trans-well assay of HT1080, LNCaP, MCF-7, NIH3T3, HCT116 and KP2 quiescent cells was performed using collagen- (Type I from rat-tail at 100 mg/ml; BD Biosciences) coated Trans-well chamber system with 8μm pore polycarbonate membrane (Nunc) as reported [7]. The cells were plated in the upper chamber at 2 x 10^4^ per well in 200 μl of phenol red-free DMEM containing 0.5% BSA (HT1080, LNCaP, MCF-7, NIH3T3 and HCT116 cells) or 200 μl of phenol red-free RPMI-1640 containing 0.5% BSA and 2 mM glutamine (KP-2 cells). Cells were allowed to migrate for 6 h in a humidified chamber at 37°C with 5% CO2 in the absence or presence of the indicated compounds. EGF was used at 100ng/ml, the synthetic androgen R1881 and DHT (Sigma), were both used at 10 nM. Casodex was used at 10 μM, and the peptides S1 and Ss were both used at 10 nM. Cells on the upper side were then detached. Cells on the underside were fixed in 4% paraformaldehyde for 15 min and stained with Hoechst 33258 for 10 min. Cells were finally counted with a DMBL (Leica) fluorescent microscope using HCPL Fluotar 20x objective. The score of migrated cells was obtained by counting ten random microscopic fields in several independent experiments, each performed in duplicate. MMP-9 activity was assayed as reported [24,25], using the fluorescent AK411 kit (BIOMOL Res. Lab.). Briefly, sub-confluent HT1080 cells were serum-starved for 24 h. Cells were left unstimulated or stimulated with EGF (100 ng/ml) for 5 min in the absence or presence of the compounds indicated in Figure 3. Casodex and the peptides (S1 or Ss) were added 30 min before growth factor stimulation. Conditioned cell culture medium was concentrated using Amicon Ultra 10K filters (Millipore, Ireland) and utilized to measure protease activity of released MMP-9 by a quenched fluorogenic peptide. MMP-9 assays were performed in a 96-well micro-plate format, according to the manufacturer’s instructions. Samples were read in a TECAN Infinite 200 fluorescent micro-plate reader, using Ex/Em=328/393. Values were obtained from several independent experiments, each performed in triplicate. 

### Immunofluorescence

Cells on coverslips were fixed and permeabilized as described [21,26]. Endogenous AR was visualized as described [7], using diluted (1:100 in PBS) rabbit polyclonal anti-AR antibody (Ab-2, Neo-Markers). Rabbit antibody was detected using diluted (1:200 in PBS containing 0.2% bovine serum albumin) anti-rabbit fluorescein-conjugated antibodies (Jackson Laboratories). The mouse monoclonal anti-BrdU antibody was detected using diluted (1:200 in PBS) Texas red-conjugated goat anti-mouse antibody (Jackson Laboratories). Coverslips were finally stained with Hoechst 33258, inverted and mounted in Mowiol (Calbiochem). Fields were analyzed with a DMBL Leica (Leica) fluorescent microscope using HCX PL Apo 63x oil and HCX PL Fluotar 100x oil objectives. Images were captured using DC480 camera (Leica) and acquired using FW4000 (Leica) software, as reported [7,26,27].

### Antibodies, immunoprecipitation, immunoblotting and Src kinase assay

Lysates (at 2 mg/ml protein concentration) were prepared as described [10]. Src tyrosine kinase was immunoprecipitated and revealed using mouse monoclonal anti-Src antibody (clone 327, from Millipore), as described [28]. The Src tyrosine kinase activity in immunocomplexes was assayed using enolase (Sigma) as a substrate [10]. AR was revealed using the rabbit polyclonal anti-AR antibodies (N-20 or C19, respectively; Santa Cruz], as described [7]. EGFR was immunoprecipitated and detected from cell lysates using the rabbit polyclonal anti-EGFR antibody (Millipore), as above described [15]. Tubulin was detected using mouse monoclonal anti-tubulin antibody (clone DM1A, Sigma). The ECL system (GE Healthcare) was used to reveal immunoreactive proteins.

### Mouse xenografts

HT1080 cells in 50% (v/v) Matrigel solution in phosphate-buffered saline (PBS; pH 7.4) were subcutaneously injected in the dorsal posterior region at 2.5 x 10^6^ cells/male athymic mice (CD mice, Charles-River Italia) without hormone priming. Animals were randomly selected for the treatment with Casodex (dissolved in 0.1% ethanol) or DHT (dissolved in 0.1% ethanol) or control vehicle intra-peritoneally for a total of 4 weeks. Each group consisted of six animals. For the treatment of each animal (average weight: 25.08 g), 200 μl of 1 μM Casodex (about 0.1 µg) in 0.1% ethanol or 200 μl of 1 μM DHT (about 3 μg/Kg body weight) or the same amount of vehicle alone were administered to the mice on alternate days. The tumor volumes of HT1080 cell xenografts were measured at indicated intervals in two dimensions by a caliper and expressed as tumor mass (mm^3^). This is calculated according to the formula: a^2^ × b × 0.5, where a is the width and b is the length of the tumor. Food and water were given ad libitum. The animals were weighed twice weekly and no difference in body weight was detected between control mice or Casodex-treated mice. Animal experimentation was reviewed and approved by the Animal Research Ethical Committee of “Istituto per la Diagnosi e la Cura dei Tumori- Fondazione “G. Pascale” (Naples-ITALY).

### Statistical analysis

As stated in the figure legends, experiments were repeated at least three times and each experimental point was measured in duplicate or triplicate. Data in Figure 1 (BrdU incorporation and tumor mass analysis) were obtained from four different experiments. For the analysis of transactivation assays, BrdU incorporation and migration, differences among values observed after the various treatments were analyzed using the Student’s t-test for paired observations. A *p* value < 0.05 was considered significant. For *in vivo* experiments, the unpaired Student’s t-test was used to compare mean values. Differences were considered statistically significant when *p* < 0.05.

## Supporting Information

Figure S1 **HT1080 cells harbor transcriptionally inactive AR.** **Androgen challenging of these cells does not induce AR nuclear translocation, but robustly increases cell motility (A-C)**. In **A**, quiescent HT1080 cells were transfected with 3416 or 3424 ARE-Luc constructs with or without hAR-expressing plasmid. Details of these procedures are described in Methods. Cells were left unstimulated or stimulated for 18 h with 10 nM R1881. Luciferase activity was assayed, normalized using beta-gal as an internal control, and expressed as fold induction. Three independent experiments were performed in triplicate Means and SEM are shown; *n* represents the number of experiments. (*) *p* value < 0,005. Inset in A shows the Western blot of HT1080 or NIH3T3 cell lysates with the rabbit polyclonal C-19 anti-AR antibody (Santa Cruz). In **B**, quiescent HT1080 cells on coverslips were left untreated or treated for 60 min with 10 nM R1881. Cells were analyzed by IF for AR (left images) or Hoechst (right images). Images are representative of 3 independent experiments. Bar, 5 μm. In **C**, NIH3T3 and HT1080 cells were left untreated or treated with 10 nM R1881, in the absence or presence of Casodex (at 10 μM). Cells were allowed to migrate for 6 h in collagen pre-coated Trans-well filters. Migrated cells were stained and counted as reported in Methods. The number of migrated cells was evaluated and expressed as relative increase. Mean and SEM are shown. n represents the number of experiments. (**) *p* value < 0,001. Uptake of fluorescein-conjugated S1 or **Ss** peptides in **HT1080** cells (**D**). In **D**, quiescent HT1080 cells on coverslips were incubated for 30 min at 4°C or 37°C with fluorescein-conjugated S1 or Ss peptide (both at 1 nM). Coverslips were analyzed by IF as described in Methods. Upper images in D show the fluorescein-conjugated S1 peptide (Fluo S1) incubated at 4°C (left image) or 37°C (right image). Lower images in D show the fluorescein-conjugated Ss peptide (Fluo Ss) incubated at 4°C (left image) or 37°C (right image). Images are representative of 3 independent experiments each performed in duplicate. Bar, 10 μm. Images in this panel show the peptides are delivered similarly into the cells. No dependence on the temperature was observed, thus excluding an energy-dependent mechanism of peptide internalization.
(TIF)

Figure S2 **NIH3T3 cells harbor transcriptionally inactive AR and androgen challenging of these cells does not induce DNA synthesis (**A**-**B**).** NIH3T3 cells were used. In **A**, cells were transfected with 3416 or 3424 ARE-Luc constructs with or without hAR-expressing plasmid and then made quiescent as reported in Methods. Cells were left unstimulated or stimulated for 18 h with 10 nM R1881. Luciferase activity was assayed, normalized using beta-gal as an internal control, and expressed as fold induction. Three independent experiments were performed in triplicate. Means and SEM are shown; *n* represents the number of experiments. (*) *p* value < 0.001. Inset in **A** shows the Western blot with rabbit polyclonal C-19 anti-AR antibody (Santa Cruz) of lysate proteins from NIH3T3 cells transfected with the pSG5 empty plasmid or transfected with pSG5 plasmid encoding the hAR. In **B**, quiescent NIH3T3 cells on coverslips were left untreated or treated for 18 h with 10 nM R1881 or EGF (100 ng/ml) or serum (20%). After *in* *vivo* labeling with BrdU (100 μM), BrdU incorporation was analyzed by IF and expressed as % of total cells. Several independent experiments were performed in duplicate and data derived from at least 700 scored cells for each coverslip. Mean and SEM are shown. n represents the number of experiments. (°) p value < 0.001. (**C**-**D**) **Casodex and S1 peptide prevent EGF-induced DNA synthesis and migration of NIH3T3 cells**. Quiescent NIH3T3 fibroblasts were used. In **C**, cells on coverslips were left unstimulated or stimulated for 18 h with the indicated compounds. EGF was used at 100 ng/ml; Casodex was used at 10 μM; S1 and Ss peptides were used at 1 nM. After *in* *vivo* pulse with BrdU (100 μM), BrdU incorporation was analyzed by IF and expressed as % of total cells. Several independent experiments were performed in duplicate and the results were derived from at least 500 scored cells for each coverslip. Mean and SEM are shown. n represents the number of experiments. Inset in **C** shows the Western blot of NIH3T3 cell lysates with the antibodies against the indicated proteins: tubulin and epidermal growth factor receptor (EGFR). The Western blot of MCF-7 cell lysate with the anti-tubulin or the anti-EGFR antibody is shown for comparison. In **D**, cells were left untreated or treated for 6 h with the indicated compounds. EGF was used at 100 ng/ml; Casodex (Cx) was used at 10 µM; both S1 and SS peptides were used at 10 nM. Cells were allowed to migrate in collagen pre-coated Trans-well filters. Migrated cells were stained and counted as reported in Methods. Results were derived from several independent experiments, each performed in duplicate. Data are expressed as relative increase. Mean and SEM are shown. n represents the number of experiments. In **C** and **D**, (**) *p* value < 0.005.
(PPTX)
