# Supplementary figures and images for: Targeting Androgen Receptor/Src Complex Impairs the Aggressive Phenotype of Human Fibrosarcoma Cells
Source: PLoS One. 2013 Oct 9;8(10):e76899. doi: 10.1371/journal.pone.0076899 (PMC3793924; doi:10.1371/journal.pone.0076899)

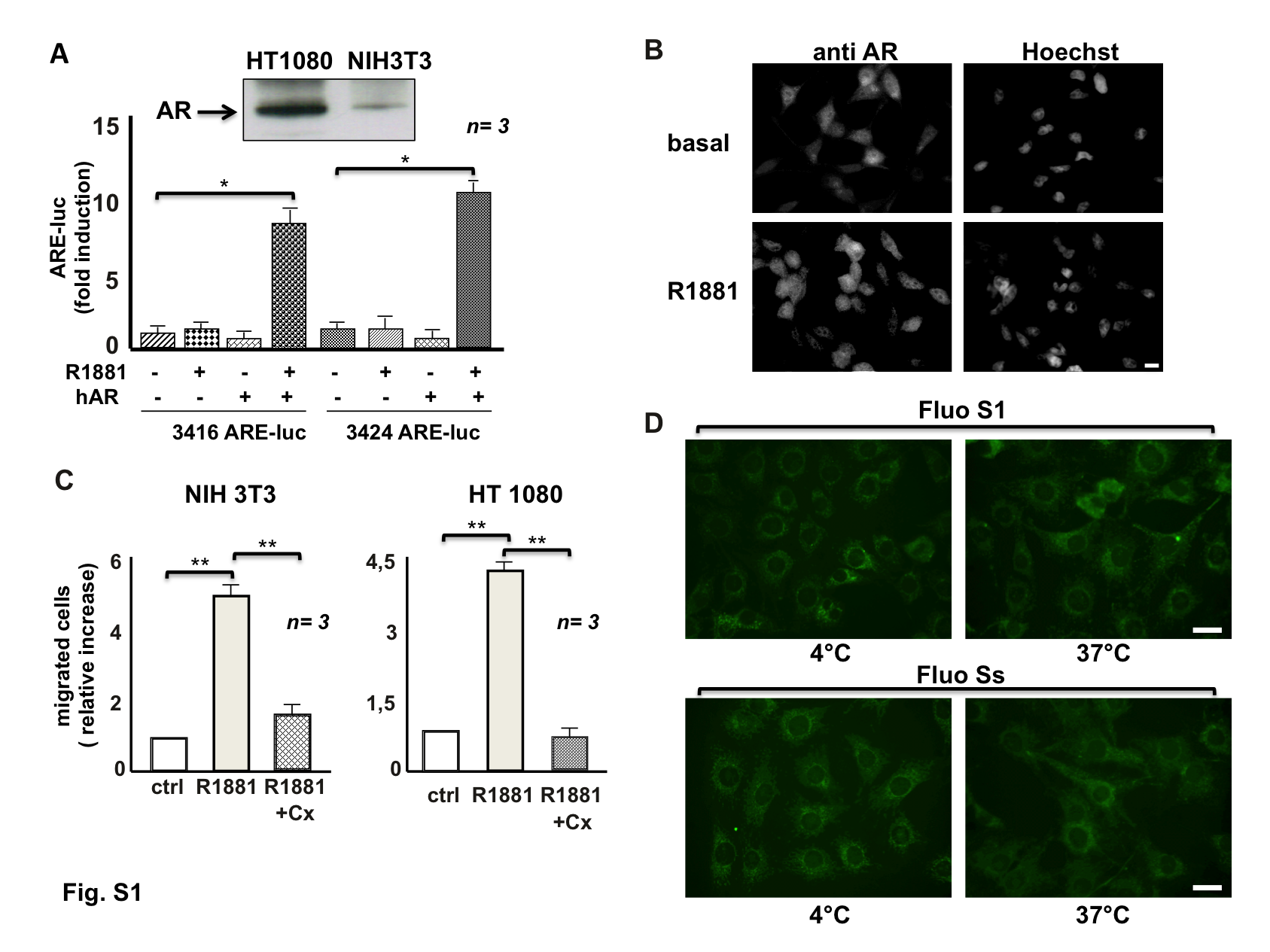

Supplement: Figure S1 — HT1080 cells harbor transcriptionally inactive AR. Androgen challenging of these cells does not induce AR nuclear translocation, but robustly increases cell motility (A-C). In A, quiescent HT1080 cells were transfected with 3416 or 3424 ARE-Luc constructs with or without hAR-expressing plasmid. Details of these procedures are described in Methods. Cells were left unstimulated or stimulated for 18 h with 10 nM R1881. Luciferase activity was assayed, normalized using beta-gal as an internal control, and expressed as fold induction. Three independent experiments were performed in triplicate Means and SEM are shown; n represents the number of experiments. (*) p value < 0,005. Inset in A shows the Western blot of HT1080 or NIH3T3 cell lysates with the rabbit polyclonal C-19 anti-AR antibody (Santa Cruz). In B, quiescent HT1080 cells on coverslips were left untreated or treated for 60 min with 10 nM R1881. Cells were analyzed by IF for AR (left images) or Hoechst (right images). Images are representative of 3 independent experiments. Bar, 5 μm. In C, NIH3T3 and HT1080 cells were left untreated or treated with 10 nM R1881, in the absence or presence of Casodex (at 10 μM). Cells were allowed to migrate for 6 h in collagen pre-coated Trans-well filters. Migrated cells were stained and counted as reported in Methods. The number of migrated cells was evaluated and expressed as relative increase. Mean and SEM are shown. n represents the number of experiments. (**) p value < 0,001. Uptake of fluorescein-conjugated S1 or Ss peptides in HT1080 cells (D). In D, quiescent HT1080 cells on coverslips were incubated for 30 min at 4°C or 37°C with fluorescein-conjugated S1 or Ss peptide (both at 1 nM). Coverslips were analyzed by IF as described in Methods. Upper images in D show the fluorescein-conjugated S1 peptide (Fluo S1) incubated at 4°C (left image) or 37°C (right image). Lower images in D show the fluorescein-conjugated Ss peptide (Fluo Ss) incubated at 4°C (left image) or 37°C [file pone.0076899.s001.tif]
