# Supplementary material for: Targeting Androgen Receptor/Src Complex Impairs the Aggressive Phenotype of Human Fibrosarcoma Cells
Source: PLoS One. 2013 Oct 9;8(10):e76899. doi: 10.1371/journal.pone.0076899 (PMC3793924; doi:10.1371/journal.pone.0076899)
Supplement: Figure S2 — NIH3T3 cells harbor transcriptionally inactive AR and androgen challenging of these cells does not induce DNA synthesis (A-B). NIH3T3 cells were used. In A, cells were transfected with 3416 or 3424 ARE-Luc constructs with or without hAR-expressing plasmid and then made quiescent as reported in Methods. Cells were left unstimulated or stimulated for 18 h with 10 nM R1881. Luciferase activity was assayed, normalized using beta-gal as an internal control, and expressed as fold induction. Three independent experiments were performed in triplicate. Means and SEM are shown; n represents the number of experiments. (*) p value < 0.001. Inset in A shows the Western blot with rabbit polyclonal C-19 anti-AR antibody (Santa Cruz) of lysate proteins from NIH3T3 cells transfected with the pSG5 empty plasmid or transfected with pSG5 plasmid encoding the hAR. In B, quiescent NIH3T3 cells on coverslips were left untreated or treated for 18 h with 10 nM R1881 or EGF (100 ng/ml) or serum (20%). After in vivo labeling with BrdU (100 μM), BrdU incorporation was analyzed by IF and expressed as % of total cells. Several independent experiments were performed in duplicate and data derived from at least 700 scored cells for each coverslip. Mean and SEM are shown. n represents the number of experiments. (°) p value < 0.001. (C-D) Casodex and S1 peptide prevent EGF-induced DNA synthesis and migration of NIH3T3 cells. Quiescent NIH3T3 fibroblasts were used. In C, cells on coverslips were left unstimulated or stimulated for 18 h with the indicated compounds. EGF was used at 100 ng/ml; Casodex was used at 10 μM; S1 and Ss peptides were used at 1 nM. After in vivo pulse with BrdU (100 μM), BrdU incorporation was analyzed by IF and expressed as % of total cells. Several independent experiments were performed in duplicate and the results were derived from at least 500 scored cells for each coverslip. Mean and SEM are shown. n represents the number of experiments. Inset in C shows the Western blot o [file pone.0076899.s002.pptx]

## Slide 1
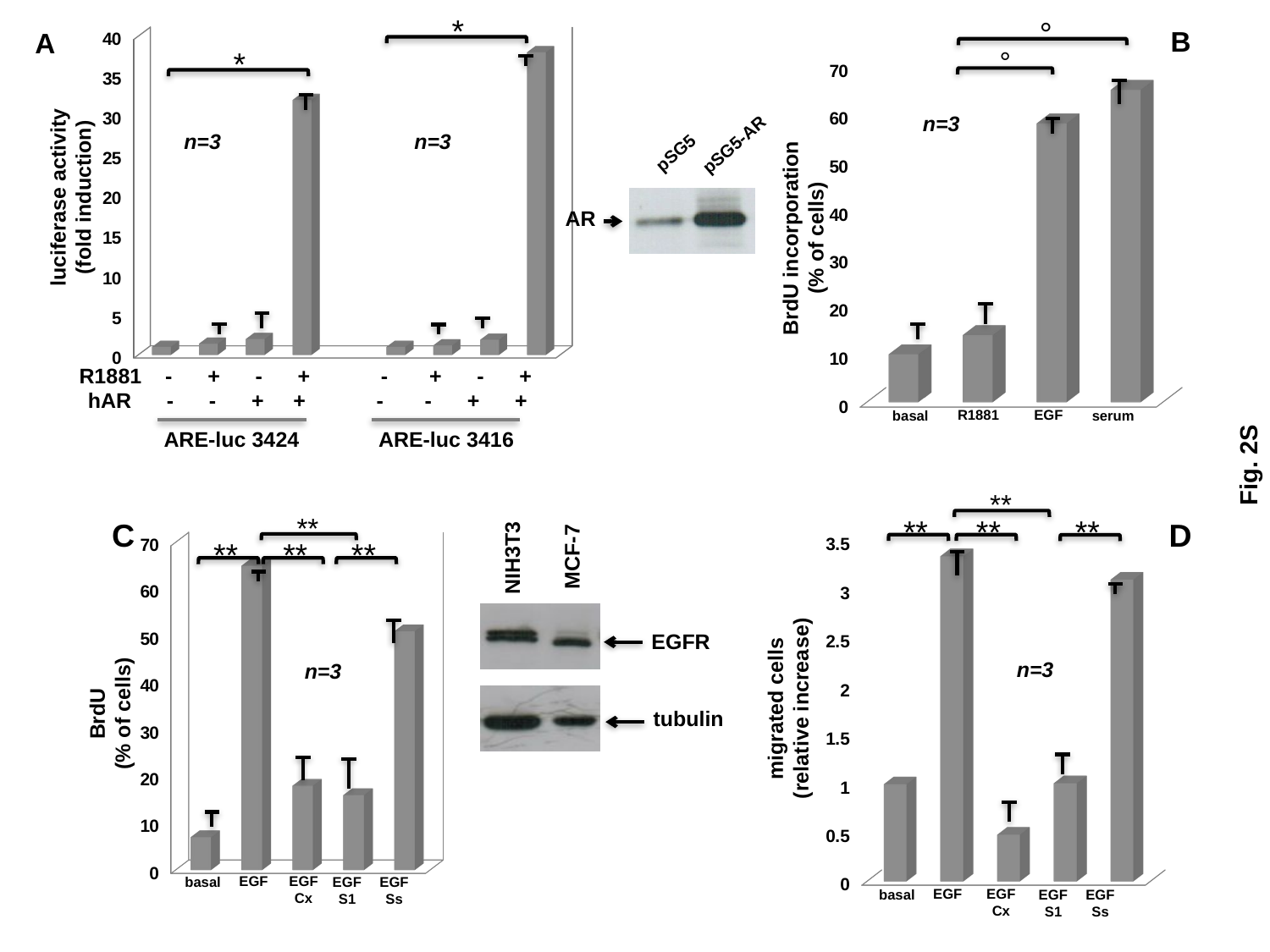

*
°
B
[unsupported chart]
A
*
°
[unsupported chart]
n=3
n=3
n=3
pSG5-AR
pSG5
luciferase activity
(fold induction)
AR
BrdU incorporation
(% of cells)
R1881 - + - + - + - +
hAR - - + + - - + +
R1881
EGF
basal
serum
ARE-luc 3424
ARE-luc 3416
Fig. 2S
**
**
**
**
**
C
D
[unsupported chart]
[unsupported chart]
**
**
**
MCF-7
NIH3T3
EGFR
n=3
n=3
migrated cells
(relative increase)
BrdU
(% of cells)
tubulin
EGF
EGF
Cx
basal
EGF
S1
EGF
Ss
EGF
EGF
Cx
basal
EGF
S1
EGF
Ss
